# Trait differences among discrete morphs of a color polymorphic lizard, *Podarcis erhardii*

**DOI:** 10.7717/peerj.10284

**Published:** 2020-11-05

**Authors:** Kinsey M. Brock, Simon Baeckens, Colin M. Donihue, José Martín, Panayiotis Pafilis, Danielle L. Edwards

**Affiliations:** 1 Department of Life & Environmental Sciences, School of Natural Sciences, University of California, Merced, Merced, CA, United States of America; 2 Quantitative and Systems Biology Graduate Group, School of Natural Sciences, University of California, Merced, Merced, CA, United States of America; 3 Laboratory of Functional Morphology, Department of Biology, University of Antwerp, Wilrijk, Belgium; 4 Department of Biology, Macquarie University, Sydney, Australia; 5 Department of Biology, Washington University in St. Louis, St. Louis, MO, United States of America; 6 Department of Evolutionary Ecology, Museo Nacional de Ciencias Naturales, CSIC, Madrid, Spain; 7 Department of Zoology and Marine Biology, National and Kapodistrian University of Athens, Panepistimioupolis, Athens, Greece; 8 Zoological Museum, National and Kapodistrian University of Athens, Athens, Greece; 9 Department of Life & Environmental Sciences, School of Natural Sciences, University of California, Merced, Merced, CA, United States of America

**Keywords:** Color polymorphism, Bite force, Chemical signals, Lizard, Traits, *Podarcis erhardii*

## Abstract

Color polymorphism defies evolutionary expectations as striking phenotypic variation is maintained within a single species. Color and other traits mediate social interactions, and stable polymorphism within a population is hypothesized to be related to correlational selection of other phenotypic traits among color morphs. Here, we report on a previously unknown throat color polymorphism in the Aegean Wall Lizard (*Podarcis erhardii*) and examine morph-correlated differences in traits important to social behavior and communication: maximum bite force capacity and chemical signal profile. We find that both sexes of *P. erhardii* have three color morphs: orange, yellow, and white. Moreover, orange males are significantly larger and tend to bite harder than yellow and white males. Although the established color polymorphism only partially matches the observed intraspecific variation in chemical signal signatures, the chemical profile of the secretions of orange males is significantly divergent from that of white males. Our findings suggest that morph colors are related to differences in traits that are crucial for social interactions and competitive ability, illustrating the need to look beyond color when studying polymorphism evolution.

## Introduction

Understanding processes that generate and maintain phenotypic variation is a fundamental goal in evolutionary biology. Color polymorphism, or the presence of multiple genetically determined color phenotypes that coexist within a breeding population, can be found in many species across the tree of life (Gray & McKinnon, 2007; Hugall & Stuart-Fox, 2012). Color polymorphic species offer a unique opportunity to study evolutionary processes underlying phenotypic variation (Ford, 1945; Huxley, 1955) such as natural and sexual selection (Kapan, 2001; Corl et al., 2010a; Seehausen, Van Alphen & Lande, 1999), gene flow (Harley et al., 2006), and genetic drift (Runemark et al., 2010), because color morphs can be used as phenotypic proxies for genetic markers (reviewed in Roulin, 2004; Svensson, 2017). However, we still have an incomplete understanding of how color polymorphism evolves, and of the evolutionary processes underlying its maintenance (reviewed in Gray & McKinnon, 2007; McLean, Stuart-Fox & Moussalli, 2014).

Defining the number of color morphs and identifying other distinct characteristics among morphs are necessary first steps in understanding evolutionary mechanisms involved in color polymorphism maintenance. Color polymorphism may vary from just two color morphs, in the case of the spiny spider *Gasteracantha fornicata* where yellow and white morphs are tuned to the local environment and coloration of sympatric flowers (Kemp et al., 2013; White et al., 2017), to systems where many color morphs occupy a wide range of habitats with varying predation pressures, such as the 20 color types in the Central American strawberry poison frog, *Oophaga pumilio* (Rudh, Rogell & Höglund, 2007; Hegna, Saporito & Donnelly, 2013). Color polymorphism may be limited to one sex (Andrés, Sánchez-Guillén & Rivera, 2000; Van Gossum, Stoks & De Bruyn, 2001; Kim et al., 2019; Moon & Kamath, 2019) or morph types may vary within and between the sexes (Sinervo, Svensson & Comendant, 2000; Martin et al., 2013), suggesting that color variants are likely under some form of sexual selection (Wellenreuther, Svensson & Hansson, 2014). In some species, color morphs indicate age or social rank (Thompson & Moore, 1991; Martin et al., 2013). Further, social interactions among morphs can dictate morph diversity across populations (Pérez i de Lanuza, Carretero & Font, 2017). Thus, the number of morphs and the maintenance of color polymorphism can be the result of natural selection, sexual selection, both natural and sexual selection, and sometimes perhaps even neutral processes (Gray & McKinnon, 2007; Rudh, Rogell & Höglund, 2007; Runemark et al., 2010; Pérez i de Lanuza, Sillero & Carretero, 2018). But across taxa, one thing remains clear: the key to understanding color polymorphism lies in identifying the number of morphs and morph-correlated characteristics and the broader context in which these alternative phenotypes are operating and interacting with each other.

Color morphs often differ in multiple traits besides color (e.g., behavioral and physiological reproductive strategies (Sinervo & Lively, 1996; Sinervo, Svensson & Comendant, 2000; Vercken & Clobert, 2008; Galeotti et al., 2013), hormone levels and immune function (Huyghe et al., 2009a; Huyghe et al., 2009b; Galeotti et al., 2010), body size (Huyghe et al., 2007), and other phenotypic characters (Lank et al., 1995)). Distinct behavioral tactics and other traits associated with different color morphs are likely the result of multivariate correlational selection for particular trait combinations (Blows & Brooks, 2003; Blows, Brooks & Kraft, 2003). Investigating morph-correlated traits is important for understanding how morphic variation is maintained, as the evolution of color polymorphism could be the result of selection on the color polymorphism itself, on a trait correlated with the color polymorphism, or suites of traits possessed by different morphs (reviewed in Gray & McKinnon, 2007). Progress has been made on morph-correlated traits and their potential role in color morph maintenance in a few well-studied systems (Sinervo & Lively, 1996; Calsbeek, Hasselquist & Clobert, 2010), but our understanding of how these traits evolve and their function in maintaining phenotypic diversity within species remains fragmented.

Lizards provide a good system to study the causes and consequences of color polymorphism because color morphs have evolved several times in squamates (e.g., Iguania: *Ctenophorus decresii*, McLean, Stuart-Fox & Moussalli, 2014; *Sceloporus grammicus*, Bastiaans et al., 2013; *Uta stansburiana,* Sinervo & Lively, 1996; *Urosaurus ornatus,* Thompson & Moore, 1991; Lacertidae: *Iberolacerta monticola* López, Moreira & Martín, 2009; *Zootoca vivipara*, Vercken & Clobert, 2008; *Podarcis gaigeae*, Runemark et al., 2010; *Podarcis muralis*, Pérez i de Lanuza et al., 2019). Most importantly, all of these lizard species share a similar color polymorphism presented as distinct color badges on the throat, suggesting a similar evolutionary origin and function (Stuart-Fox & Ord, 2004). Color polymorphism is common among lacertid lizards, and the lacertid genus *Podarcis* is highly color polymorphic (Sacchi et al., 2007; Huyghe et al., 2007; Runemark et al., 2010; Pérez i de Lanuza, Sillero & Carretero, 2018). Previous studies of color polymorphic *Podarcis* species have identified color morph differences in size and survival (Calsbeek, Hasselquist & Clobert, 2010), ability to win staged contests (Huyghe et al., 2012; Abalos et al., 2016), absolute maximum bite force capacity and head muscle mass (Huyghe et al., 2008), and circulating hormone levels associated with aggressive behaviors (Huyghe et al., 2009a; Huyghe et al., 2009b). A lizard’s body size and bite force capacity are critical functional traits that directly relate to its ability to acquire and defend resources (Huyghe et al., 2005; Donihue et al., 2015). In color polymorphic *Podarcis* species, male morphs differ in their maximum bite force capacity (Huyghe et al., 2007; Pérez i de Lanuza & Font, 2014), largely due to morph differences in head size (Huyghe et al., 2009a; Huyghe et al., 2009b). Color morphs may have different head morphologies due to different levels of circulating hormone levels, such as testosterone, associated with muscle development (Huyghe et al., 2009b; Regnier & Herrera, 1993). Morphs may also differ in head size and bite force as a consequence of partitioning their dietary niche (Lattanzio & Miles, 2016), as differences in diet hardness can influence lizard bite force among species and even populations (Herrel et al., 1999; Herrel, Vanhooydonck & Van Damme, 2004; Donihue, 2016). In lizards, male size and bite force are important determinants in the outcome of agonistic and mating encounters and can indicate overall male quality (Tokarz, 1985; Lailvaux et al., 2004; Huyghe et al., 2005; Hardy & Briffa, 2013). Thus, color morph differences in bite force can play a significant role in an individual’s social status.

In addition to visual signals such as color, chemical signals also play an important role in intra-specific communication and social organization in reptiles (Mason & Parker, 2010; Martín & López, 2014; Baeckens, 2019). In lacertids, male lizards have distinct femoral glands that produce a lipid- and protein-rich exudate (López & Martín, 2002; López & Martín, 2002; Mayerl, Baeckens & Van Damme, 2015; Mangiacotti et al., 2017). Both males and females strongly rely on chemical cues and use information from these secretions, particularly from the lipophilic fraction (Martín & López, 2015), to select mates (López & Martín, 2002), judge competitive ability (Carazo, Font & Desfilis, 2007) and dominance status (López & Martín, 2002). In some lacertid species, discrete male color morphs are also chemically polymorphic in waxy secretions exuded from their femoral pores (Pellitteri-Rosa et al., 2014; Mangiacotti et al., 2019a). This suggests morph-correlated chemical signals may be used to indicate male quality and health status and be involved in mate choice. These discoveries suggest sexually selected traits such as size, bite force, and chemical communication are important for social interactions that may influence morph fitness (López, Moreira & Martín, 2009; Lopez-Darias et al., 2015; Pérez i de Lanuza, Carretero & Font, 2017). Thus, we postulate these traits mediating social interactions are likely important to the evolutionary maintenance of color polymorphism in lizards.

The Aegean wall lizard (*Podarcis erhardii*) has been widely studied ecologically (Pafilis et al., 2009; Brock et al., 2015; Lymberakis et al., 2018), but remains an understudied species when it comes to color polymorphism, though it displays variation in throat color within populations (Arnold & Burton, 1978). To date, there have been no studies on the variable throat colors displayed by *P. erhardii*. Here we undertake the first study examining color morphs in this species. We examined the relationships among color, bite force, and chemical signal profile in a large island population of lizards which exhibit variation in throat colors. Specifically, we investigated whether throat color can be reliably discriminated into three discrete color morphs, and if color morphs differ in two traits important to lizard social behavior: maximum bite force and chemical profiles from exudate secreted from male femoral pores. Our main questions and predictions are:

(1) Do the throat color patches on adult *P. erhardii* represent discrete color morphs?

Given previous work in other *Podarcis* species (e.g., Huyghe et al., 2007; Calsbeek, Hasselquist & Clobert, 2010; Andrade et al., 2019), we expect that variation in *P. erhardii* throat color is discrete and can be discriminated into three morph types: orange, yellow, and white, and that both sexes contain the same three color morphs.

(2) Do color morphs differ in their maximum bite force capacity?

We postulate that color morphs will have different maximum bite force capacities and associated differences in morphological traits important to lizard bite force such as body size and head morphometrics.

(3) Do male color morphs differ in their chemical signal profile?

Given previous work on chemical signatures from other color polymorphic lacertids (López, Moreira & Martín, 2009; Pellitteri-Rosa et al., 2014; Mangiacotti et al., 2019a), we expect that *P. erhardii* morphs benefit from conveying information on their life strategies (or polymorphism) not only through color, but also through scent. Therefore, we predict that polymorphism in coloration matches polymorphism in chemical signal profile in *P. erhardii*.

## Material and Methods

### Study species

**Figure 1:**
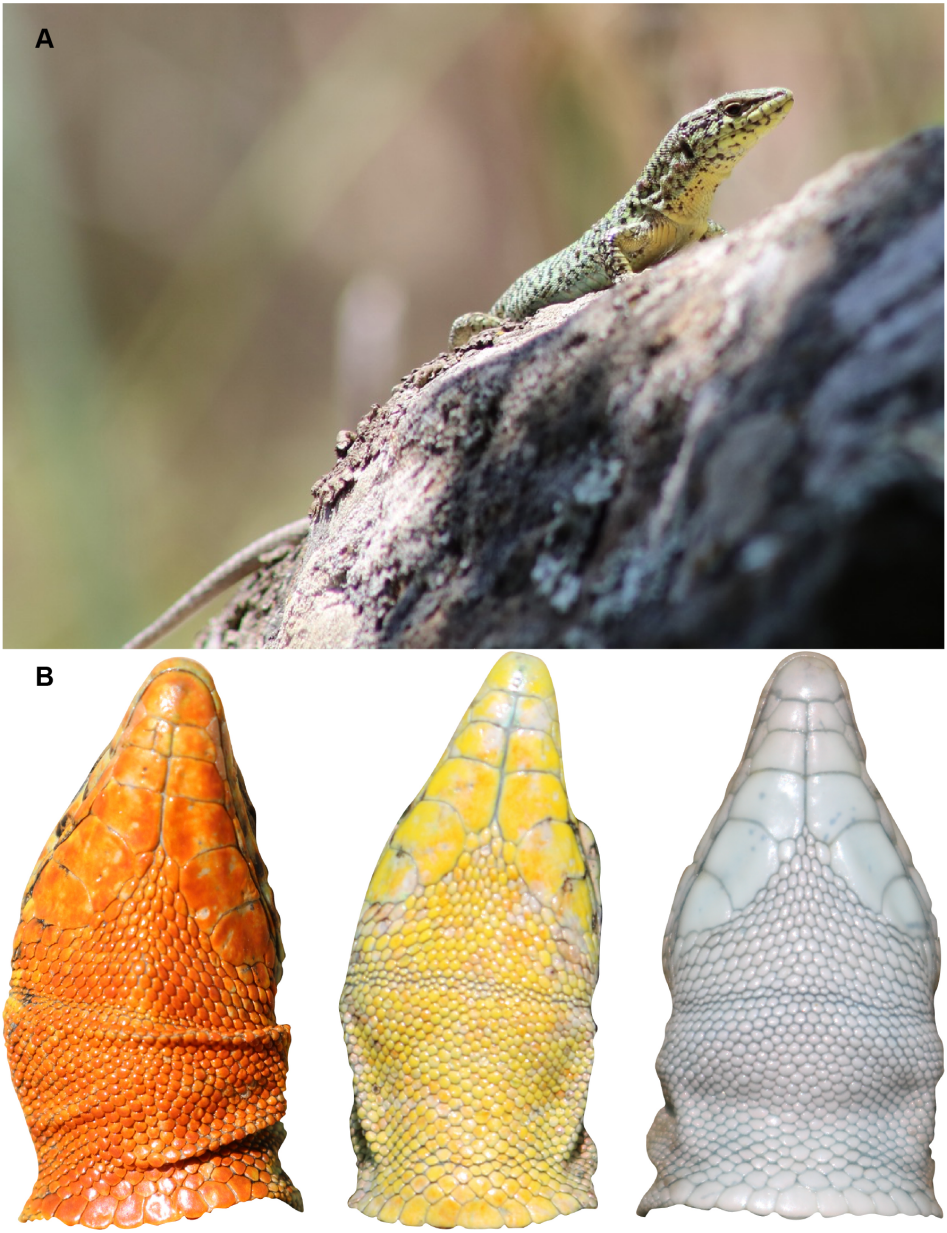
Study species, *Podarcis erhardii*. (A) An adult male yellow morph *P. erhardii* basking on a dry stone wall in Moni, Naxos, Greece. (B) Throat colors of adult male *P. erhardii* from our study population in Moni, Naxos, Greece. In *P. erhardii*, color polymorphism is restricted to the throat region and the rest of the venter is usually white.

The Aegean Wall Lizard (*Podarcis erhardii*) is a small to medium-sized ground-dwelling lacertid with an adult snout-vent length (SVL) of 45–78 mm and a tail approximately twice as long as the body (Gruber, 1987, Fig. 1A). This species exhibits substantial variation in dorsal color, pattern, body size, morphology, and behavior across its range (Brock et al., 2015; Donihue et al., 2015; Marshall et al., 2015). Similar to other color polymorphic *Podarcis* species (Andrade et al., 2019), *Podarcis erhardii* is known to have variable throat coloration within populations (Arnold & Burton, 1978; Fig. 1B), and displays geographic variation in the frequency of throat color morphs across island and mainland populations (KM Brock, 2019, unpublished data). This species of wall lizard is endemic to the southern Balkans and occurs on hundreds of Aegean islands (Valakos et al., 2008; Speybroeck et al., 2016). True to their vernacular name, they are typically encountered in habitats that are a mixture of dry stone walls surrounded by a mixture of low, spiny phrygana vegetation and grasses.

### Study area

We conducted our study in the terraced agricultural village of Moni (elev. 590 m a.s.l., 37°04′54.1″N, 25°29′35.0″E) in the foothills below Profitis Ilias peak on Naxos island. Naxos is the largest island (440 km^2^) in the Cyclades island cluster (Aegean Sea, Greece) and harbors *P. erhardii* from low elevation sandy substrates with sparse vegetation to mid-elevation montane landscapes with diverse dwarf scrub communities. We chose Moni village because the site is largely representative of habitats on the island. The site is situated off a remote hiking path lined with dry stone walls that run through terraced agricultural plots. Vegetation at the site is a mixed matrix of grasses, sclerophyllous evergreen maquis, phrygana (*Euphorbia acanthothamnos*), and olive trees (*Olea europaea*).

### Field methods

Adult *P. erhardii* were captured from our study site in Moni during the month of May in 2017 and 2018. Lizards collected in 2017 include both adult females and males, while lizards in 2018 consist of only males used for exudate chemical analysis. To avoid sampling the same males from 2017 in 2018, we only used individuals with complete un-autotomized tails as all lizards from 2017 had 20 mm of tail tip taken for specimen collection. Upon capture, we sexed lizards; adult males were determined from their enlarged femoral pores and swollen tail base, and adult females were distinguishable by their smaller heads, longer bodies relative to head size, and absence of femoral pores. Lizards with a SVL less than 45 mm were deemed immature and inappropriate to include in color and morphometric comparisons, and immediately returned to their capture site. Animals were transported from the field to the laboratory in individual cloth lizard bags for subsequent measurement. All research was conducted in accordance with the University of California, Merced Institutional Animal Care and Use Committee (IACUC protocol AUP17-0002) and permits provided by the Greek Ministry for Environment and Energy (codes Ψ4Γ64653Π8-HΛ5 and Ω8Δ84653Π 8-BΞX assigned to KM Brock).

### Throat color measurements and analysis

We collected lizard throat color data from 47 females (*N* = 8 orange, 21 yellow, and 18 white individuals) and 85 males (*N* = 31 orange, 29 yellow, and 25 white individuals) in May 2017 (2017 lizards were also used in bite force analyses). To quantify color, lizard throat color patches were first measured with an Ocean Optics Flame S-UV–VIS Fibre Optic Spectrometer 200–850 nm (Ocean Optics Inc. Dunedin, FL, USA) and Xenon pulse light source connected to a probe with a fibre optic cable. Measurements were calibrated with a white WS-1-SL Labsphere Diffuse Reflectance Standard (Spectralon, Ocean Optics). Spectra were collected by placing the spectrometer illumination probe perpendicular to the surface of the throat 5 mm away from the skin (Pérez i De Lanuza, Font & Carazo, 2013; Badiane et al., 2017). Measurements were a circular point sample three mm × three mm, and we took six measurements of lizard throat color patches at landmark throat scale locations to avoid measurement bias and capture potential variation across the entire signal (Pérez i de Lanuza et al., 2019).

Lizards were visually examined by K.M. Brock and assigned to one of three throat color categories, or morphs: orange, yellow, or white. Throat color signals in this species are comparatively simple and clear to discriminate by eye (Fig. 1B) compared to other species that have mottling or fine throat color patterning (Teasdale, Stevens & Stuart-Fox, 2013). For each lizard, we averaged the six throat spectra from the throat patch using the *aggspec* function in the R package ‘pavo’ to calculate one spectral measurement per lizard for analysis (Maia et al., 2019). Averaged spectra were then smoothed to reduce noise in our reflectance curves (Teasdale, Stevens & Stuart-Fox, 2013). To determine our smoothing parameter, we assessed reflectance curves against our raw, unsmoothed curves and set our smoothing span option to 0.2 nm to preserve shape and minimize noise. We extracted a suite of 23 colorimetric variables pertaining to hue, saturation, and brightness from our smoothed total reflectance data using the *rspec* function in the ‘pavo’ package in R (Montgomerie, 2006; Maia et al., 2019). We selected 10 of the extracted colorimetric variables (mean brightness, intensity, UV chroma, yellow chroma, green chroma, blue chroma, red chroma, contrast, and hue; Supplemental Information 1) for their relevance to the trichromatic visual system of wall lizards (Martin et al., 2015), robustness to smoothing correction (Maia et al., 2019) and satisfaction of collinearity assumptions of K-means clustering and Linear Discriminant Function Analysis (LDFA) analyses. These 10 variables were used in all of our color analyses.

We first determined the optimal number of color morph categories in each sex we used an unsupervised K-means clustering analysis. We used the *clusGap* function in the R package ‘cluster’ (v.2.0.7-1, Maechler et al., 2019), which calculates a goodness of clustering measure with a gap statistic (Tibshirani, Guenther & Hastie, 2002). The gap statistic uses the output from the K-means clustering algorithm and compares the total within-cluster dispersion for different values of K with their expected values under a null reference distribution of the data (Tibshirani, Guenther & Hastie, 2002). The estimated optimal number of clusters is where the gap statistic is maximized, or furthest away from a random uniform distribution of points. We *a priori* set the potential number of K-means to 6, given the number of color morphs in other *Podarcis* species (Huyghe et al., 2007; Calsbeek, Hasselquist & Clobert, 2010; Runemark et al., 2010; Andrade et al., 2019).

Finally, we used a Linear Discriminant Function Analysis to predict color morph groupings from our 10 colorimetric predictor variables and assure accuracy of our visual assignments using the *lda* function from the ‘MASS’ package (v 7.3-50) in R (Venables & Ripley, 2002). LDFA determines group means of the 10 colorimetric predictor variables for each morph and computes, for each individual, the probability of belonging to different color morph categories. We then tested if color morphs significantly differed based on the same 10 colorimetric variables using a Wilk’s Lambda test.

For both the K-means clustering analysis and LDFA, we analyzed females and males caught in 2017 separately to reliably identify morph types for each sex. All analyses and data visualization were performed in R (v. 1.1.456) (R Core Team, 2018).

### Bite force measurements and analysis

We collected bite force data from adult females and males in May 2017. These same lizards were used in the color analyses mentioned above. We measured lizard bite force with a purpose-built bite force meter consisting of two metal bite plates connected to a Kistler force transductor (type 9203; Kistler Inc., Switzerland) and pivot over a microcaliper fulcrum (full bite force meter specifications in Herrel et al., 1999). Bite plates were placed toward the anterior of the lizards’ mouth in straight alignment with the lizards’ body to ensure consistent replication across individuals (Lappin & Jones, 2014). We recorded bite force of each individual in three repeated trials and used the hardest bite as our measure of maximum bite force (Anderson, Mcbrayer & Herrel, 2008; Donihue et al., 2015). Lizard size is known to positively correlate with bite force capacity in this system (Donihue et al., 2015), so we also measured several lizard head and body features that may explain variation in bite force among morphs. Morphometric data taken for each lizard include: SVL, head length (tip of snout to posterior of parietal scale), head width (at the widest point just posterior of ear opening), and head height (at posterior of parietal scale). Lizard morphometric data used in bite force analyses were taken with a Mitutoyo 500-171-30 Absolute Scale Digital Caliper.

Maximum bite force is known to differ substantially between the sexes across many taxa for social and ecological reasons (Herrel, Van Damme & De Vree, 1996; Herrel, McBrayer & Larson, 2007; Sagonas et al., 2014; Donihue, 2016). Keeping this pattern in mind, we analyzed the sexes separately (*N* = 45 females, *N* = 81 males). We assessed bite force data for normality and subsequently removed 4 outliers from the dataset that were well below the minimum first quartile, most likely due to a poor grip on the bite plate. Bite force and morphometric data were normally distributed and did not require transformation for analysis. We used ANOVAs and post hoc Tukey HSD tests to investigate univariate differences in bite force related head traits among morphs. To evaluate the influence of SVL and color morph on bite force, we ran Generalized Linear Models (GLMs, Dobson, 1990) with maximum bite force as the dependent variable and SVL and color morph as independent variables. GLMs were run using the *glm* function in the ‘stats’ package (v3.5.1) in R (R Core Team, 2018). All analyses and data visualization were carried out in R (v. 1.1.456) (R Core Team, 2018).

### Collection of glandular secretions and chemical analysis

In 2018, we re-visited our study population in Moni and collected glandular secretions and corresponding throat color measurements from 39 adult lizards (*N* = 11 orange males, *N* = 15 yellow males, and *N* = 13 white males). Since the femoral glands of females are vestigial and non-active (Mayerl, Baeckens & Van Damme, 2015), only males were sampled. Immediately after the lizards were captured, we collected femoral gland secretions by gently squeezing around the femoral pores. The secretions were subsequently transferred to glass vials with glass inserts sealed with Teflon-lined lids. Blank controls were also created to exclude any contaminants from the handling procedure or the environment and to examine potential impurities in the solvent or analytical procedure. All vials were, thereafter, stored at −20 °C before chemical analysis.

The identification of each chemical compound and estimation of its relative abundance (as percentage) was assessed using gas-chromatography-mass spectrometry (GC-MS). Here, we used the same methodology and protocol as described in earlier studies (e.g., López, Moreira & Martín, 2009; López, Moreira & Martín, 2009; Baeckens et al., 2018; Supplemental Information 2). Because we were interested in examining differences among different color morphs in the overall chemical profile, we determined the relative amount of each compound as the percent of the total ion current (TIC) as in García-Roa et al. (2018).

Prior to statistical analyses of chemical profiles, proportions were logit transformed by taking the natural logarithm of proportion / (1-proportion). A constant value (0.01) was added to eliminate zero values in the data set allowing logit transformation. This compositional analysis corrects for the non-independece of proportions (Aebischer, Robertson & Kenward, 1993). To test for differences in the chemical profiles of lizards belonging to different color morphs, we performed a single factor permutational multivariate analysis of variance (PERMANOVA, Anderson, 2001; McArdle & Anderson, 2001). To do so, we first calculated Euclidean distances between every pair of individual samples to produce a resemblance matrix that formed the basis of the PERMANOVA (set at 999 permutations). To assess among-morph differences in more detail, we investigated the chemical profiles further using a canonical analysis of principal coordinates (CAP, Anderson & Willis, 2003) and a principal component analysis (PCA). Next, we performed univariate analyses of variance (ANOVAs) to test for inter-morph differences in chemical profiles based on principal component scores of the first few principal axes. We also tested for body size-dependent variation in the chemical signal design of *P. erhardii* by regressing SVL against the scores of the principal components. Data were analyzed in R v3.6.1 (R Core Team, 2018) and the software PRIMER v6.1.13 (Clarke & Gorley, 2006) with the ‘PERMANOVA’ +v1.0.3 add-on package.

## Results

An unsupervised K-means cluster analyses of 10 colorimetric variables (Table 1) revealed that the optimal number of color clusters for both females and males is 3 (Table 2). For both females and males, the global optimal number of K-means was 3, with a maximum gap statistic at K-means = 3 (Table 2).

**Table 1:** Results from linear discriminant function analyses testing for the presence of three discrete color morphs in *Podarcis erhardii*. Coefficients of linear discriminants are shown for each of the 10 colorimetric variables used in LDFA of females from 2017 (*N* = 47), males from 2017 (*N* = 85).

	2017 Females *N* = 47		2017 Males *N* = 85	
Linear discriminant	Linear discriminant 1	Linear discriminant 2	Linear discriminant 1	Linear discriminant 2
Mean brightness	0.092	0.138	−0.213	0.088
Intensity	0.105	0.156	0.101	0.051
UV chroma	−1318.082	11529.59	−867.469	5969.393
Yellow chroma	133.069	−63.721	−12.332	−105.373
Blue chroma	−1299.786	11522.04	−846.666	5934.362
Green chroma	−1355.169	11550.36	−888.693	6035.815
Red chroma	−1395.448	11597.21	−887.764	6007.215
Contrast	−0.155	−0.246	0.049	−0.105
Spectral saturation	−6.105	−2.555	−9.871	0.476
Hue	0.129	0.041	−0.019	0.027

**Table 2:** Observed vs. predicted frequencies of color morph category assignments from linear discriminant function analyses. For 2017 females and males, our models including 10 colorimetric variables predicted all observed yellow individuals to belong to the yellow morph category. Observed orange individuals were never predicted to be white, and observed white individuals were never predicted to be orange. In a few instances, observed orange and white individuals were predicted to belong to the yellow morph category.

		Observed orange	Observed yellow	Observed white	Sum
2017 Females	Predicted orange	7	0	0	7
	Predicted yellow	1	20	1	22
	Predicted white	0	0	18	18
	Sum	8	20	19	47
2017 Males	Predicted orange	27	0	0	27
	Predicted yellow	4	29	1	34
	Predicted white	0	0	24	24
	Sum	31	29	25	85

**Figure 2:**
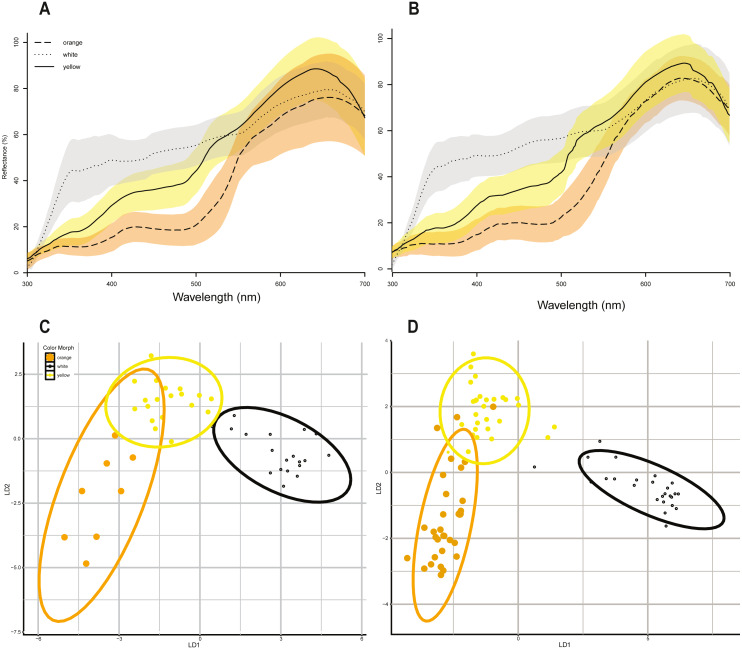
Color morphs in *Podarcis erhardii*. (A) Smoothed spectral reflectance curves of P. erhardii female color morphs. (B) Smoothed spectral reflectance curves of P. erhardii male color morphs. The average reflectance curves in (A) and (B) for orange morphs is represented by a long-dashed line, the average for yellow morphs is represented by a solid line, and the average for white morphs is a dotted line. (C) Linear discriminant function analysis of morphs from female color data. (D) Linear discriminant function analysis of morphs from male color data. For females and males, the first linear discriminant function (*x*-axis, LDA1) separates orange and yellow morphs from white morphs, with no overlap in the 95% confidence interval of white morphs. The second linear discriminant function (*y*-axis, LDA2) separates orange and yellow morphs, with some overlap in both females and males.

Linear discriminant function analyses of the same 10 colorimetric variables (Table 1) discriminated between the three color morph classes (Figs. 2A & 2B) in *P. erhardii* with some overlap in the 95% confidence ellipses of orange and yellow morphs for both sexes (Figs. 2C & 2D). Results from LDFA of females and males show that the three color morphs are discernable (Figs. 2C & 2D), and color morphs significantly differ based on the 10 colorimetric variables (Wilks’ Lambda *p* < 0.001). Morph predictions from LDFAs for both females and males closely matched observed morph assignments (Table 3). For females, multivariate discrimination of color morph based on the 10 colorimetric variables was significant (Wilks’ Lambda = 0.042, F(2,44) = 13.533, *p* < 0.001). For males, multivariate discrimination of color morph based on the same 10 colorimetric variables was also significant (Wilks’ Lamdba = 0.029, F(2,82) = 35.729, *p* < 0.001).

**Table 3:** Gap statistic results from K-means cluster analysis. For both females and males, the optimal number of clusters in the colorimetric data was 3. Maximized gap statistic, where K-clusters are maximally distant from each other, is indicated in bold.

	2017 Females *N* = 47		2017 Males *N* = 85	
K-means	Gap statistic	Std. Error	Gap statistic	Std. Error
1	0.272	0.035	0.309	0.025
2	0.363	0.033	0.440	0.024
3	**0.385**	0.031	**0.502**	0.022
4	0.358	0.031	0.492	0.022
5	0.366	0.032	0.496	0.021
6	0.382	0.032	0.495	0.021

**Figure 3:**
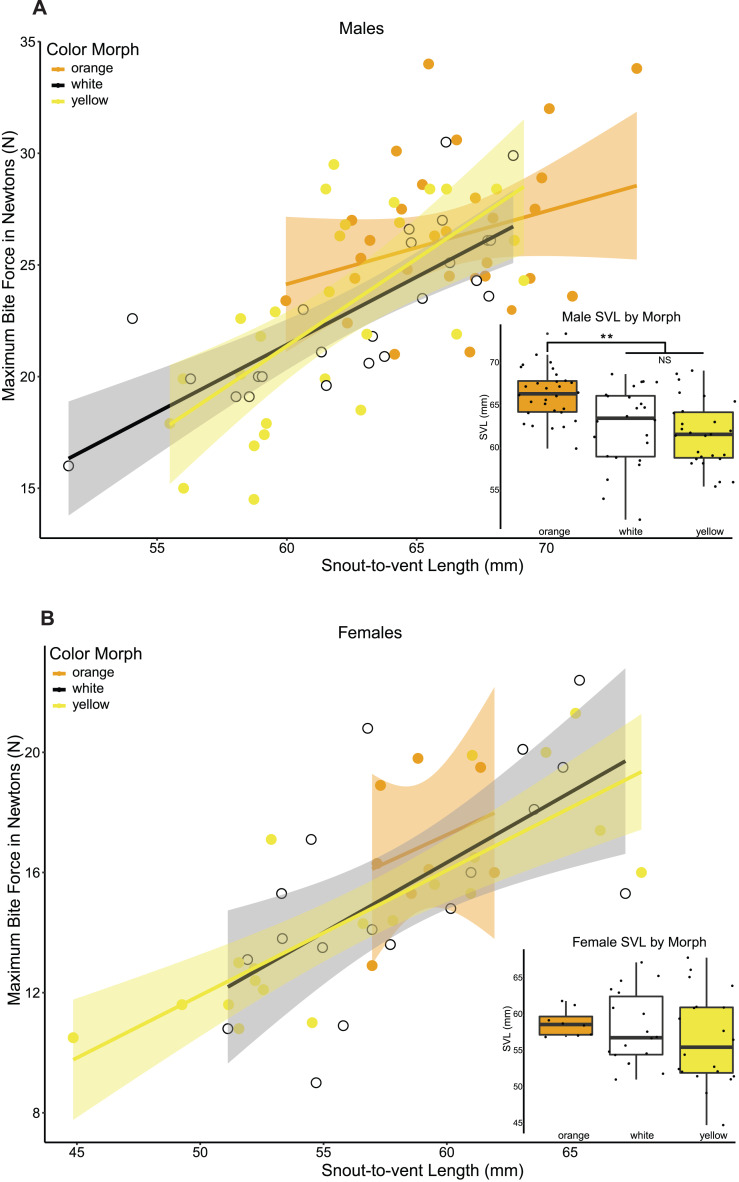
Relationship between maximum bite force and lizard body size (SVL) among color morphs. (A) Relationship between SVL and maximum bite force capacity in male color morphs. Bite force had a positive relationship with body size across all male color morphs. Within morphs, a significant positive correlation between maximum bite force and SVL was detected for yellow (Pearson corr = 0.663, *R*^2^ adj = 0.417) and white (Pearson corr = 0.781, *R*^2^ adj = 0.593, *p* < 0.001) morphs, while no significant correlation was detected for orange morphs (Pearson corr = 0.282, lm *R*^2^ adj = 0.047, *p* = 0.13). Boxplot of male snout-to-vent length (SVL) by color morph. Orange males had significantly longer SVLs than white and yellow males (ANOVA SVL morph F(2,78) = 11.6, *p* < 0.001, Tukey HSD orange-white *p* = 0.001, orange-yellow *p* < 0.001, denoted by a double asterisk). (B) Relationship between SVL and maximum bite force capacity in female color morphs. Bite force also increased with body size across all female color morphs. We detected a significant positive relationship between maximum bite force capacity and SVL for yellow (Pearson corr = 0.808, lm *R*^2^ adj = 0.634, *p* < 0.001) and white (Pearson corr = 0.637, lm *R*^2^ adj = 0.369, *p* < 0.005) morphs. The relationship between bite force capacity and SVL was positive but not statistically significant for orange females (Pearson corr = 0.298, lm *R*^2^ adj = −0.063, *p* = 0.473). We did not detect a significant difference in SVL among female color morphs.

Maximum bite force was strongly positively correlated with all head morphometrics (head length, head width, head height) and head morphometrics were also strongly positively correlated with body size in both sexes (Table 4), so we focus here on the relationship between bite force, color morph, and body size because body size drives head size. Maximum bite force in male lizards was strongly positively correlated with lizard body size (Linear regression maximum bite force ∼SVL, adj. *r*^2^ = 0.644, *p* = 0.002, *N* = 81, *df* = 79, Fig. 3A), and male color morphs had significantly different SVLs (ANOVA SVL ∼morph F(2,78) = 11.6, *p* < 0.05, Table 4, Fig. 3A). A post hoc Tukey HSD test showed that orange males had significantly longer SVLs than white and yellow males, which did not differ from each other in SVL (Table 4). We then analyzed differences in bite force among male morphs that accounted for lizard size in a GLM that included morph and SVL as fixed effects, and found no significant effect of morph on maximum bite force capacity (GLM maximum bite force ∼morph + SVL, F(3,77) = 13.41, morph *p* > 0.05, SVL *p* < 0.001).

**Table 4:** ANOVA and Tukey HSD tests of SVL and head morphometric variables among color morphs. Females and males were analyzed separately. Bolded values with an asterisk denotes a significant difference in size between color morphs detected at alpha = 0.05.

Morphometric ANOVA	Post hoc comparisons	Male Difference	Male *P (adj)*	Female Difference	Female *P (adj)*
Male SVL F(2,78) = 11.6 Female SVL F(2,43) = 0.527	white-orange	−3.748	0.001*	−0.805	0.932
	yellow-orange	−4.601	<0.001*	−2.268	0.568
	yellow-white	−0.853	0.709	−1.463	0.675
Male head length F(2,78) = 14.64 Female head length F(2,43) = 0.221	white-orange	−1.161	<0.001*	−0.158	0.892
	yellow-orange	−1.33	<0.001*	−0.523	0.285
	yellow-white	−0.169	0.821	−0.365	0.360
Male head width F(2,78) = 12.41 Female head width F(2,43) = 0.469	white-orange	−0.865	<0.001*	−0.062	0.957
	yellow-orange	−0.879	<0.001*	−0.184	0.669
	yellow-white	−0.014	0.997	−0.123	0.744
Male head height F(2,78) = 11.1 Female head height F(2,43) = 0.822	white-orange	−0.417	0.043*	−0.287	0.431
	yellow-orange	−0.422	0.032 *	−0.245	0.531
	yellow-white	−0.005	0.999	−0.043	0.968

Female maximum bite force was also positively correlated with body size (Linear regression maximum bite force ∼SVL, adj. *r* ^2^ = 0.704, *p* < 0.001, *N* = 46, *df* = 44, Fig. 3B). In contrast to males, female color morphs did not differ in body size (ANOVA SVL ∼morph F(2,43) = 0.651, *p* = 0.527, Fig. 3B,da Table 4), or head morphometrics (Table 4). In a GLM that included morph and SVL as fixed effects, we did not detect a significant effect of morph on maximum bite force for female color morphs of *P. erhardii* from the same population (GLM maximum bite force ∼morph + SVL, F(3,42) = 14.72, morph *p* > 0.05, SVL *p* < 0.001).

From the femoral gland secretions of male *P. erhardii* (*N* = 39) originating from the study population in Moni (Naxos, Greece), we could identify 81 different lipophilic compounds (Supplemental Information 3). Considering all individuals together, secretions were mainly a mixture of steroids (average ± SE % of TIC: 68.4 ±1.21%), waxy esters (17.7 ± 0.82%) and tocopherol (5.6 ±0.88%). Fatty acids (3.3 ± 0.40%), alcohols (1.8 ± 0.10%), and aldehydes (1.4 ± 0.11%) were present in intermediate concentrations. Amides (0.6 ± 0.13%), ketones (0.5 ± 0.06%), a terpenoid (0.3 ± 0.06%), and furanones (0.3 ± 0.03%) were the four chemical classes with the lowest average proportion. On average, the five most abundant chemicals were cholesterol (39.8 ± 1.37%), campesterol (7.9 ± 0.41%), *α*-tocopherol (5.6 ± 0.88%), the 1,2-ethanediyl ester of hexadecanoic acid (5.5 ± 0.47%), and *β*-sitosterol (4.2 ± 0.45%).

**Figure 4:**
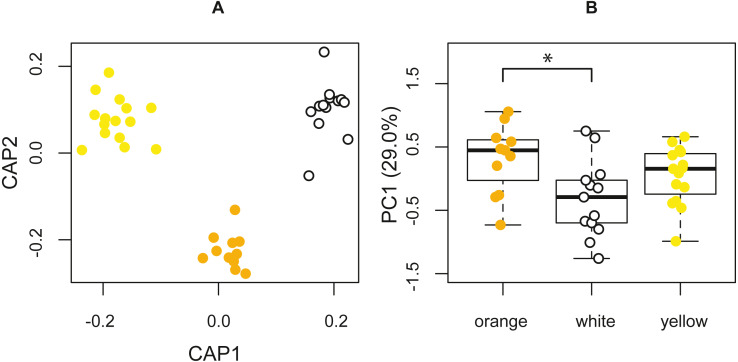
Chemical analyses of pore exudate. (A) A representation of the two first axes of the canonical analysis of principal coordinates (CAP) showing classification of the chemical profile of the three *P. erhardii* color morphs. (B) A boxplot showing inter-morph differences in PC1 scores. Asterisk annotates statistical significance.

The PERMANOVA, based on the resemblance matrix comparing the three morphs, showed borderline significant differences in the chemical profile of the three color morphs (pseudo F(2,38) = 1.587, *p* = 0.067). Pairwise PERMANOVA tests indicated statistically significant differences in the chemical composition of the glandular secretions, specifically, between orange and white morphs (*t* = 1.590, *p* = 0.021). There were no significant differences between yellow and white morphs (*t* = 1.125, *p* = 0.243) and orange and yellow morphs (*t* = 0.991, *p* = 0.435). The CAP analysis classified 51.28% of the chemical profiles into the correct population using leave-one-out cross-validation (*δ* = 0.97, *p* = 0.389, *m* = 34 axes; Fig. 4A).

The first four principal components jointly explained 64.0% of the variation, with the first axis (29.0%) being strongly affected by *α*-tocopherol (loading = 0.70), eicosyl 9-octadecenoate (0.27), the 1,2-ethanediyl ester of hexadecanoic acid (−0.25), octadecanoic acid (0.25), and tetradecyl 9-octadecenoate (−0.24). We used the scores of the first four principal components (PCs) to test for among-morph differences using four separate univariate analyses of variance (ANOVAs). These tests indicated that PC1 varied significantly among color morphs (F(2,36) = 4.273, *p* = 0.0216; Fig. 4B). A post hoc LSD multi-comparison (with Bonferroni correction) showed a significant difference between white and orange color morphs (Table 5), with orange males having higher proportions of *α*-tocopherol, eicosyl 9-octadecenoate, and octadecanoic acid, but lower proportions of 1,2-ethanediyl ester of hexadecanoic acid and tetradecyl 9-octadecenoate. There were no significant among-morph differences in PC2, PC3, and PC4 (all F ≤ 0.669; p ≥ 0.518). Lastly, variation in the chemical composition of the glandular secretion of *P. erhardii* could not be explained by variation in body size as none of the PCs showed a significant link with SVL (all F ≤ 1.382, p ≥ 0.175).

## Discussion

Prior to this study, *P. erhardii* was known to have variable throat color (Arnold & Burton, 1978; Runemark et al., 2010), but its status as a color polymorphic species was never recognized as the number and types of color morphs were unknown. Multiple species in the *Podarcis* genus are color polymorphic (Andrade et al., 2019), and *P. erhardii* can now be counted among them. We used quantitative analyses of spectral reflectance data to reliably classify *P. erhardii* into three discrete color morphs: orange, yellow, and white. We have also established that both sexes have the same three color morphs, which is not always the case even among species of color polymorphic lizards (e.g.: *Ctenophorus decresii* (Rankin et al., 2016), *Urosaurus ornatus* (Hews & Moore, 1995), *Uta stansburiana* (Sinervo & Lively, 1996; Sinervo, Svensson & Comendant, 2000). Determining the number and types of morphs within color polymorphic species is an essential first step toward understanding the evolution and maintenance color polymorphism, and identifying morph-associated traits relevant to fitness is the logical next step toward that goal.

**Table 5:** A post hoc LSD multi-comparison (with Bonferroni correction) among male morphs for PC1. A significant difference detected (*p* < 0.05) is bolded with an asterisk.

Post hoc comparisons of 2018 male morph chemical profiles	Difference	*P (adj)*
orange - white	0.629	0.019*
orange - yellow	0.265	0.649
white - yellow	−0.364	0.237

In some color polymorphic species, morph color can indicate age or social rank (Thompson & Moore, 1991; Martin et al., 2013), and thus individuals may have the ability to change morph over their lifetime (Carpenter, 1995). In a color polymorphic Phrynosomatid lizard, *Urosaurus ornatus*, females retain an orange throat color badge into adulthood while males tend to experience ontogenetic throat color change from orange to blue, suggesting some social function (Carpenter, 1995). In our study population, orange males tend to be larger than yellow and white males, and it is possible that male color morphs exhibit ontogenetic color change or experience different growth rates. A recent study on the genomic basis of throat color polymorphism in closely related *P. muralis* found that throat color is controlled by genetic differences at two small regulatory regions of the genome, is heritable, and shared by seven species across the *Podarcis* clade (Andrade et al., 2019). The evolutionary maintenance of color polymorphism across the *Podarcis* clade seems to be the result of retained ancient genetic variation and hybridization (Andrade et al., 2019). We do not yet know if *P. erhardii* shares the same genomic architecture as other color polymorphic *Podarcis* species, or if temporary ontogenetic shifts can alter morph state (Andrade et al., 2019). Further research is needed that incorporates skeletochronological assessment of age and long-term monitoring of individual color *in situ* to fully understand the underlying mechanisms that control throat color in this species and the entire *Podarcis* clade.

A lizard’s bite force is directly related to its ability to acquire and defend essential resources for survival and reproduction (Huyghe et al., 2005), such as food (Verwaijen, Van Damme & Herrel, 2002; Huyghe et al., 2007), territory (Husak et al., 2006), and mates (Vitt & CooperJr, 1985). We predicted that distinct color morphs would have different maximum bite force capacities. We found that body size varied substantially between male color morphs, with orange males exhibiting significantly larger body and head size than yellow and white males, while female morphs did not significantly differ in and size dimensions. When controlling for body size, we do not observe a difference in bite force between color morphs in either sex. The proximate driver of variation in bite force is body size, and orange males tend to be larger than yellow or white males and thus have relatively stronger bites. Our results are comparable to findings from closely related *P. melisellensis* (Huyghe et al., 2009a; Huyghe et al., 2009b), where orange males have larger head dimensions and also bite significantly harder than yellow and white males. It is well-established that body size and bite force matters in determining fight outcomes in male lizards (Vitt & CooperJr, 1985; Anderson & Vitt, 1990; Sacchi et al., 2009). Further, male-biased sexual size dimorphism is also indicative of sexual selection for larger male size, either through intra-sexual selection via male-male competition over territory and mates or inter-sexual selection through female mate choice (Shine, 1989). Given that *P. erhardii* males have larger heads and body size than females, and male color morphs differ in their size and bite force capacity, it is plausible that male morphs use different strategies for survival and reproduction. Alternative color morph strategies specifically related to fitness are common, and likely play a role in balancing selection that maintain morphs through time, such as the different behavioral reproductive tactics employed by male morphs that generate differences in access to mates in *Uta stansburiana* (Sinervo & Lively, 1996). Interestingly, we did not find the same pattern in female color morphs, which exhibited no difference in body size dimensions or bite force capacity. Few studies on color morph differences in morphology, performance, behavior, and physiology include females (Sinervo & Lively, 1996; Huyghe et al., 2009a; Huyghe et al., 2009b; Bastiaans et al., 2013), which combined with long-held assumptions about female reproductive behavior has hindered our understanding of underlying mechanisms of evolution (Kamath & Losos, 2017). An interesting and open question is whether female color morphs differ in their reproductive strategies and behaviors (Vercken & Clobert, 2008; Galeotti et al., 2013; Ortega et al., 2015), particularly if they have a preference for males based on color (Pérezi De Lanuza, Font & Carazo, 2013), morph-correlated traits, or both. Future study on *P. erhardii* color morph reproductive strategies and their effects on fitness are needed to provide insight into mechanisms that balance and maintain color polymorphism.

Our findings show that the chemical composition of the glandular secretions of *P. erhardii* males varies among color morphs, and that this variation is body size-independent. Although the established polymorphism in discrete colors only partly matched the observed intraspecific variation in chemical signal signatures (as the chemical profiles of yellow males could not be distinguished statistically from white or orange males), the chemical profile of the secretions of orange males did significantly differ from those of white males. While comparable findings on color polymorphism partially matching chemical polymorphism have been reported for two other lacertid species, *Iberolacerta monticola* (two color morphs; López, Moreira & Martín, 2009) and *Podarcis muralis* (three color morphs; Pellitteri-Rosa et al., 2014; Mangiacotti et al., 2019a), to our knowledge no such findings have been reported in any other color polymorphic vertebrate taxa.

One hypothesis on why animals would benefit from broadcasting their individual morph state (and potentially alternative strategies) in different ways is to enhance signal effectiveness (redundant signaling hypothesis; (Johnstone, 1996; Partan, 2013). The use of different signal modalities, both visual and chemical, might increase the chance that the message is accurately perceived by the receiver. For instance, including scent marks in ones signaling repertoire could be beneficial as they work in darkness and can remain operative in the absence of signaler (Müller-Schwarze, 2006). Yet, our findings show an incomplete overlap in visual and chemical polymorphism. One potential reason for this is methodological. We took a multivariate approach in our analyses to statistically test for morph differences. However, it could also be that lizards may discriminate between color morphs solely based on the absence or presence of a single compound, on the absolute concentration of a specific chemical, or on a specific combination of molecules (Wyatt, 2014). Alternatively, the visual and chemical signals of *P. erhardii* may not be redundant, but may convey different information to the receiver (multiple signaling hypothesis, Hebets & Papaj, 2005). For instance, in another lacertid lizard, *Lacerta schreiberi*, large color patches on the body of adult males and the chemical composition of their femoral secretions provide different information on the individual’s levels of carotenoids and vitamin E (Kopena, López & Martín, 2014). As such, exploiting a bimodal signaling system allows animals to convey multiple messages at once. More elaborate behavioral research is necessary to find out (1) whether color morph-specific differences in chemical signatures are functional, in that lizards can discriminate based on scent alone, and (2) whether lizards can discriminate color morphs on the multicomponent profile of the femoral secretions or if discrimination is based solely on individual compounds and changes in their proportions. In addition, since recent findings suggest that also the proteinic fraction of femoral secretions can play a role in lizard intraspecific communication (Mangiacotti et al., 2017; Mangiacotti et al., 2019a; Mangiacotti et al., 2019b; Mangiacotti et al., 2019c), future studies that combine proteinic and lipophilic assessments of chemical signals should be encouraged.

Our results show that orange males tend to have higher proportions of *α*-tocopherol, eicosyl 9-octadecenoate, and octadecanoic acid, but lower proportions of the 1,2-ethanediyl ester of hexadecanoic acid and tetradecyl 9-octadecenoate than the other two color morphs. Previous research has shown that in lacertids, *α*-tocopherol (= vitamin E) and octadecanoic acid are two important compounds for intraspecific communication, with the relative proportions of lipids in the femoral gland secretions providing information on overall male quality (Martín & Lopez, 2014; Martín & Lopez, 2015). Several studies have found that *α*-tocopherol can be exploited as an honest sexual signal, with high proportions of the compound increasing the attractiveness of a male’s scent to female conspecifics (Kopena et al., 2011; García-Roa et al., 2017). Our finding that orange males produce secretions with high proportions of *α*-tocopherol (which indicate ‘high quality’ in other lacertid males) are consistent with our finding that orange males are the largest morph with the hardest relative bite force in comparison to the other two morphs. Additionally, Martín et al. (2007, 2008) showed that octadecanoic acid can act as a chemical ornament in lacertids signaling individual health, with lizards in good health (indicated by a high T-cell-mediated immune response) having low proportion of octadecanoic acids in their secretions. In this study, we observe that orange *P. erhardii* males secrete high proportions of octadecanoic acid, which partially goes against our earlier argument on orange males being the morph in ‘best condition’. Still, it might be that the low immune response and associated high proportion of secreted octadecanoic acid is the result of orange males having high levels of testosterone. This is not unlikely since the immunosuppressive effect of testosterone is well documented in lizards (e.g., Belliure, Smith & Sorci, 2004; Oppliger et al., 2004), and so is the observation that color morphs experience dissimilar hormone levels (e.g., Sinervo, Svensson & Comendant, 2000; Huyghe et al., 2009a; Huyghe et al., 2009b; Sacchi et al., 2017). Chemical signals from males may interact with other male morph-correlated traits and behaviors, as well as female preference to balance the relative fitness of color morphs through time. Endocrinological and immunological research is required to determine whether *P. erhardii* color morphs differ in hormone levels and immunocompetence, and whether these may cause among-morph differences in chemical signal profile and fitness.

## Conclusion

We found that *P. erhardii* male color morphs tend to differ in two important lizard social traits: their maximum bite force capacities (driven by body size differences) and chemical signal signatures. These observations suggest that male differences in physical and chemical traits may play an important role in color polymorphism maintenance via social interactions. Further investigation into male morph competitive ability and female morph mate preference will shed light on how these traits relate to sexual signaling and morph persistence. Throat color polymorphism has been associated with sexual selection in lizards (Sinervo & Lively, 1996; Sinervo & Calsbeek, 2006). Competition between males over females often results in male-biased sexual size dimorphism and male attributes that signal status, fighting ability, and fitness (Enquist & Leimar, 1983; Andersson, 1994). It is currently unknown whether *P. erhardii* prefers the scent of certain morphs with certain qualities, or whether there is assortative mating based on color, size, scent preference, or behavior. Future work that integrates color morph behavior, reproductive strategies, and phenotypic traits is needed to determine the role of social traits in the maintenance of color polymorphism.

##  Supplemental Information

10.7717/peerj.10284/supp-1 Supplemental Information 1 Colorimetric variables extracted from smoothed spectral reflectance curves used in linear discriminant function analyses

10.7717/peerj.10284/supp-2 Supplemental Information 2 Chemical data generation

10.7717/peerj.10284/supp-3 Supplemental Information 3 Lipophilic compounds found in femoral gland secretions of male *Podarcis erhardii* lizards of three different color morphs

10.7717/peerj.10284/supp-4 Supplemental Information 4 Code for all analyses

10.7717/peerj.10284/supp-5 Supplemental Information 5 Data for all analyses

## Additional Information and Declarations

### Field Study Permissions

The following information was supplied relating to field study approvals (i.e., approving body and any reference numbers): Greek Ministry for Environment and Energy approved the study (codes Ψ4Γ64653Π8-HΛ5 and Ω8Δ84653Π8-BΞX).

### Data Availability

The following information was supplied regarding data availability: Raw data and code are available in the Supplemental Files.
