# Supplementary material for: Trait differences among discrete morphs of a color polymorphic lizard, Podarcis erhardii
Source: PeerJ. 2020 Nov 5;8:e10284. doi: 10.7717/peerj.10284 (PMC7649010; doi:10.7717/peerj.10284)
Supplement: Supplemental Information 1 [file peerj-08-10284-s001.pdf]

Supplemental 1 Colorimetric variables extracted from smoothed spectral reflectance curves used in linear discriminant function analyses. Descriptions are based on values extracted using the ‘pavo’ package (Maia et al., 2019) for color analysis in R and Montgomerie (2006). All colorimetric data extracted and analyzed for each individual are labeled with the shorthand metric indicators ‘B2’, ‘B3’, and so on.

**Metric**	**Description**
**‘B2’ Mean brightness**	Mean relative reflectance over the entire spectral range.
**‘B3’ Intensity**	Maximum relative reflectance - reflectance at wavelength of maximum reflectance.
**‘S1U’ Chroma UV**	Relative contribution of UV spectral range to the total brightness (UV: 300-400nm)
**‘S1Y’ Chroma Yellow**	Relative contribution of yellow spectral range to the total brightness (Yellow: 550nm-625nm)
**‘S1G’ Chroma Green**	Relative contribution of green spectral range to the total brightness (Green: 510nm-605nm)
**‘S1B’ Chroma Blue**	Relative contribution of blue spectral range to the total brightness (Blue: 400nm-510nm)
**‘S1R’ Chroma Red**	Relative contribution of red spectral range to the total brightness (Red: 605nm-700nm)
**‘S6’ Contrast**	Reflectance maximum - Reflectance minimum
**‘S7’ Spectral saturation**	Difference between the relative reflectance before and after the wavelength at which reflectance is halfway between its minimum and its maximum
**‘H1’ Peak wavelength hue**	Wavelength of maximum reflectance
