# Supplementary material for: Trait differences among discrete morphs of a color polymorphic lizard, Podarcis erhardii
Source: PeerJ. 2020 Nov 5;8:e10284. doi: 10.7717/peerj.10284 (PMC7649010; doi:10.7717/peerj.10284)
Supplement: Supplemental Information 2 [file peerj-08-10284-s002.pdf]

Supplemental 2 We analyzed the samples using an Agilent 7890A gas chromatograph (GC) (Agilent Technologies, Palo Alto, CA, USA) fitted with a poly (5% diphenyl/95% dimethylsiloxane) column (HP5-MS, 30 m length x 0.25 mm ID, 0.25 mm film thickness) coupled to an Agilent 5975C Triple Axis Detector mass spectrometer (MS) operated in electron impact ionization mode (EI, 70 eV of electron energy). The current of the filament was 150 µA. We performed splitless sample injections (1 µl of each sample dissolved in 100 µl n-hexane) with helium as the carrier gas at a constant flow rate of 30 cm/s, and injector and detector temperatures at 250 °C and 280 °C, respectively. The oven temperature program started at 45 °C, was maintained isothermal for 10 min, then increased to 280 °C at a rate of 5 °C/min, and finally isothermal (280 °C) for 15 min. Mass spectral fragments below m/z = 46 were not recorded. Initial identification of secretion components was done by comparing their mass spectra with those in the NIST/EPA/NIH (NIST 02) computerized mass spectral library. We confirmed identifications by comparing spectra and retention times with those of authentic standards (from Sigma-Aldrich Chemical Co.) when these were available. Impurities identified in the control vial samples were not considered. Because we were interested in examining differences among different color-morphs in the overall chemical profile, we determined the relative amount of each compound as the percent of the total ion current (TIC) as in García-Roa et al., (2018).
