# Supplementary material for: Trait differences among discrete morphs of a color polymorphic lizard, Podarcis erhardii
Source: PeerJ. 2020 Nov 5;8:e10284. doi: 10.7717/peerj.10284 (PMC7649010; doi:10.7717/peerj.10284)
Supplement: Supplemental Information 3 [file peerj-08-10284-s003.pdf]

Supplemental 3 Lipophilic compounds found in femoral gland secretions of male *Podarcis erhardii* lizards of three different color morphs. The relative amount of each component was determined as the percent of the total ion current (TIC) and reported as the average (±SE). Characteristic ions (m/z) are reported for unidentified ("Unid.") compounds. RT: Retention time. An asterisk after the compound name denotes that the identification of this compound was confirmed with standards, while the rest were tentative identifications based on mass spectra comparisons.

RT	Compound	Orange (n=11)			White (n=13)			Yellow (n=15)		
**27.0**	Tetradecanal *	0.05	±	0.01	0.08	±	0.01	0.07	±	0.01
**28.8**	Tetradecanol *	0.12	±	0.02	0.17	±	0.03	0.13	±	0.01
**28.9**	2-Pentadecanone *	0.04	±	0.01	0.03	±	0.01	0.03	±	0.01
**29.3**	Pentadecanal *	0.01	±	0.01	0.01	±	0.01	0.01	±	0.01
**31.0**	A tetradecenal	0.01	±	0.01	0.03	±	0.01	0.02	±	0.01
**31.4**	Hexadecanal *	0.58	±	0.14	1.28	±	0.10	0.94	±	0.11
**32.7**	An hexadecenal	0.03	±	0.01	0.02	±	0.01	0.03	±	0.01
**33.1**	2-Heptadecanone *	0.26	±	0.02	0.29	±	0.03	0.27	±	0.02
**33.5**	Heptadecanal	0.03	±	0.01	0.03	±	0.01	0.03	±	0.01
**33.6**	Hexadecanoic acid, methyl ester *	0.06	±	0.02	0.04	±	0.01	0.02	±	0.01
**34.3**	Hexadecanoic acid *	1.07	±	0.54	0.18	±	0.08	0.30	±	0.10
**35.0**	Hexadecenol	0.01	±	0.01	0.01	±	0.01	0.01	±	0.01
**35.4**	Octadecanal *	0.16	±	0.05	0.36	±	0.03	0.26	±	0.03

**37.0**	2-Nonadecanone *	0.39	±	0.15	0.09	±	0.02	0.30	±	0.09
**37.6**	Octadecanol *	0.97	±	0.15	0.87	±	0.12	0.75	±	0.12
**37.9**	9-Octadecenoic acid *	0.48	±	0.14	0.24	±	0.12	0.32	±	0.08
**38.1**	Octadecanoic acid *	3.13	±	0.59	1.78	±	0.46	2.59	±	0.50
**38.8**	Nonadecanol	0.03	±	0.02	0.20	±	0.11	0.04	±	0.01
**39.1**	An octadecenal	0.04	±	0.02	0.06	±	0.01	0.06	±	0.01
**40.7**	Dihydro-5-tetradecyl-2(3H)-furanone	0.13	±	0.02	0.13	±	0.02	0.17	±	0.02
**41.1**	Eicosanol *	0.62	±	0.07	0.55	±	0.10	0.49	±	0.03
**42.2**	Docosanol *	0.03	±	0.01	0.04	±	0.01	0.03	±	0.01
**43.5**	9-Octadecenamide *	0.07	±	0.04	0.04	±	0.01	0.02	±	0.01
**44.1**	Unidentified furanone	0.10	±	0.05	0.13	±	0.05	0.11	±	0.04
**44.7**	Tetracosanol *	0.03	±	0.01	0.05	±	0.01	0.04	±	0.02
**47.5**	Hexacosanol *	0.10	±	0.05	0.10	±	0.04	0.11	±	0.05
**47.7**	13-Docosenamide *	0.37	±	0.13	0.75	±	0.31	0.54	±	0.18
**48.5**	Squalene *	0.33	±	0.13	0.36	±	0.11	0.29	±	0.06
**49.0**	Cholesta-3,5-diene *	0.04	±	0.01	0.05	±	0.01	0.04	±	0.01
**49.2**	Cholesta-4,6-dien-3-ol *	0.03	±	0.01	0.05	±	0.01	0.05	±	0.01
**49.3**	Unid. steroid (141,156,209,349,364)	0.09	±	0.02	0.04	±	0.01	0.06	±	0.01

**49.4**	Cholesta-3,5-diene, unid. derivative?	0.11	±	0.02	0.12	±	0.01	0.12	±	0.02
**49.8**	Unid. steroid (155,197,251,349,364)	0.45	±	0.06	0.27	±	0.03	0.38	±	0.05
**50.0**	Cholesta-5,7,9(11)-trien-3-ol	0.21	±	0.03	0.18	±	0.07	0.16	±	0.02
**50.3**	Unid. steroid (143,156,351,366)	0.17	±	0.04	0.13	±	0.01	0.16	±	0.02
**50.3**	Unid. steroid (327,379,392)	0.10	±	0.04	0.10	±	0.01	0.08	±	0.02
**50.4**	Unid. steroid (251,341,376,400,430)	0.02	±	0.01	0.01	±	0.01	0.02	±	0.01
**50.7**	Unid. steroid (141,156,341,363,378)	0.22	±	0.03	0.17	±	0.01	0.23	±	0.02
**51.0**	Unid. steroid (199,253,331,357,379)	0.05	±	0.01	0.06	±	0.01	0.05	±	0.01
**51.1**	Unid. steroid (197,199,251,361,376)	0.07	±	0.01	0.05	±	0.01	0.05	±	0.01
**51.2**	Unid. steroid (197,251,363,378)	0.69	±	0.06	0.48	±	0.04	0.67	±	0.06
**51.5**	Unid. steroid (195,209,363,378)	0.19	±	0.02	0.12	±	0.01	0.18	±	0.02
**51.6**	Unid. steroid (195,251,363,378)	0.11	±	0.03	0.07	±	0.01	0.08	±	0.01
**51.7**	Unid. steroid (143,158,183,195,253,364,380)	0.24	±	0.02	0.16	±	0.02	0.24	±	0.02
**51.8**	Unid. steroid (141,156,209,382,392)	0.28	±	0.03	0.30	±	0.04	0.32	±	0.03
**52.0**	Tetradecyl 9-octadecenoate *	0.17	±	0.04	1.70	±	0.51	0.69	±	0.19
**52.4**	Unid. steroid (197,251,377,392)	0.81	±	0.08	0.67	±	0.08	0.87	±	0.08
**52.5**	Cholesterol *	36.33	±	2.67	40.59	±	2.89	41.75	±	1.54

**52.5**	α-Tocopherol *	8.05	±	2.16	4.08	±	1.49	5.03	±	0.94
**52.9**	Unid. steroid (183,195,378,394)	0.50	±	0.04	0.68	±	0.11	0.63	±	0.05
**54.0**	Campesterol *	8.16	±	0.77	7.37	±	0.73	8.17	±	0.68
**54.1**	Cholest-4-en-3-one *	1.45	±	0.15	1.70	±	0.19	1.32	±	0.13
**54.7**	Ergost-7-en-3-ol. *	0.35	±	0.18	0.10	±	0.03	0.18	±	0.04
**55.0**	Lanosta-8.24-dien-3-one	0.10	±	0.05	0.05	±	0.01	0.05	±	0.02
**55.0**	Ergosta-5.8-dien-3-ol *	0.01	±	0.01	0.07	±	0.05	0.14	±	0.10
**55.2**	Unid. steroid (339,365,398)	0.22	±	0.20	0.04	±	0.03	0.06	±	0.05
**55.4**	Unid. Steroid (267,365,380,396,414)	2.38	±	0.53	2.48	±	0.54	2.18	±	0.45
**55.6**	Hexadeceyl hexadecanoate *	0.96	±	0.28	1.10	±	0.28	0.83	±	0.25
**55.7**	β-Sitosterol *	4.16	±	0.87	4.34	±	0.91	4.03	±	0.63
**55.8**	Cholest-5-en-3-one *		-			-		0.37	±	0.25
**56.3**	4,4-Dimethyl-cholesta-8,14-dien-3-ol *	1.46	±	0.49	1.97	±	0.63	2.44	±	0.75
**56.3**	Unid. steroid (267,379,394)	0.02	±	0.02	0.01	±	0.01	0.01	±	0.01
**56.4**	4,4-Dimethyl-cholesta-5,7-dien-3-ol *l	0.49	±	0.07	0.32	±	0.07	0.40	±	0.05
**56.6**	Cholestane-3,6-dione *	0.03	±	0.01	0.04	±	0.01	0.02	±	0.01
**57.0**	Unid. steroid (283,311,393,453)	0.39	±	0.15	0.43	±	0.10	0.41	±	0.09
**57.1**	Unid. steroid (267,377,392)	0.02	±	0.01	0.01	±	0.01	0.01	±	0.01

**57.3**	Unid. steroid (214,267,379,394)	1.44	±	0.13	1.10	±	0.11	1.40	±	0.11
**57.4**	Stigmast-4-en-3-one *	0.02	±	0.02	0.08	±	0.04	0.04	±	0.03
**58.1**	Stigmasta-3,5-dien-7-one *	0.65	±	0.07	0.52	±	0.05	0.63	±	0.06
**59.1**	Unid. steroid (214,267,393,408)	2.53	±	0.18	2.77	±	0.25	2.63	±	0.16
**59.4**	Octadecyl hexadecanoate *	3.12	±	0.66	2.00	±	0.30	2.18	±	0.36
**59.6**	Unid. steroid (356,471,486)	0.29	±	0.05	0.27	±	0.05	0.35	±	0.06
**60.3**	Unid. waxy ester (211,239,267)	0.24	±	0.04	0.19	±	0.03	0.28	±	0.02
**60.7**	Unid. steroid (191,209,291,318)	0.09	±	0.02	0.12	±	0.02	0.21	±	0.08
**61.2**	Unid. waxy ester (255,283)	1.12	±	0.26	1.27	±	0.18	0.70	±	0.10
**61.8**	Unid. waxy ester (243,257,523)	0.23	±	0.06	0.18	±	0.03	0.16	±	0.02
**63.6**	Octadecyl 9-octadecenoate *	0.27	±	0.05	0.96	±	0.28	0.48	±	0.10
**64.2**	Eicosyl 9-octadecenoate	3.76	±	0.97	1.87	±	0.34	2.23	±	0.49
**64.8**	Eicosyl hexadecanoate	3.15	±	0.63	3.50	±	0.25	3.41	±	0.47
**66.8**	Octadecanoic acid, ethenyl ester	0.07	±	0.02	0.05	±	0.01	0.09	±	0.02
**67.4**	Hexadecanoic acid. 1,2-ethanediyl ester	4.60	±	0.92	7.06	±	0.81	4.74	±	0.64
